# Associated Risk Factors with Low Back Pain in White-Collar Workers—A Cross-Sectional Study

**DOI:** 10.3390/jcm11051275

**Published:** 2022-02-25

**Authors:** Urszula Żywień, Katarzyna Barczyk-Pawelec, Tomasz Sipko

**Affiliations:** Department of Physiotherapy, University School of Physical Education in Wroclaw, al. Ignacego Jana Paderewskiego 35, 51-612 Wroclaw, Poland; ula.zywien@gmail.com (U.Ż.); katarzyna.barczyk-pawelec@awf.wroc.pl (K.B.-P.)

**Keywords:** spine, lower back pain, posture, spinal curvatures, pain thresholds

## Abstract

Objective: The purpose of the study was to compare the pressure pain threshold (PPT) of soft tissue and the curvatures of the spine in a sitting position and to estimate associated physical risk factors with low back pain (LBP) in young adults. Subjects: White-collar workers (*n*= 139), both women (*n* = 51) and men (*n* = 88) were separated into a control group (*n* = 82) and a low-intensity LBP (NRS < 3) (*n* = 57). Methods: The PPTs were tested utilizing the Wagner algometer. The curvatures of the spine were measured employing the photogrammetric method. In the logistic regression model, the odds ratio (OR) was estimated with ±95% confidence interval (CI) indicating the probability of the reported LBP. Results: The PPTs of soft tissue (OR = 1.1; CI = 1.02–1.19; *p* < 0.05) and the angle of the thoracolumbar spine in the everyday, habitual sitting position (OR = 1.19; CI = 1.05–1.34; *p* < 0.05) were associated with low-intensity LBP in female subjects. Additionally, the low intensity LBP were associated with the angles of the torso (OR = 1.14; CI = 1.01–1.29; *p* < 0.05) and the lumbosacral spine in the corrected sitting position (OR = 1.06; CI = 0.98–1.15; *p* > 0.05) and BMI (OR = 1.56; CI = 0.84–2.90; *p* > 0.05) in male subjects. Conclusion: Individual risk factors were associated with the low-intensity LBP only in females utilizing the PPT and the thoracolumbar angle in the habitual sitting position study factors. Men from the LBP group did not effectively correct the lumbosacral angle. Therefore, re-educated, self-corrected posture with specific postural training would be expected to improve proprioception in postural control capacity and result in decreasing pain.

## 1. Introduction

The number of people in Europe and the USA suffering from spinal pain is reaching 49% to 70% of the entire adult population [1]. The prevalence of low back pain was higher among women than men across all age groups. The mean difference was significant between those aged 20–29 and those aged 40–69 [2]. Low back pain (LBP) was defined as pain involving or derived from structures in the lumbosacral region between the lower posterior margin of the rib cage and the horizontal gluteal fold and chronic low back pain as present at least 12 h per day and lasting more than six months. This definition allows for intermittent/recurring or constant pain [3].

In the etiopathogenetic course of LBP, LBP is prevalent in 37% of the entire population. There are different sources of back pain, including arthritis and disc degeneration, which can initiate inflammatory symptoms, with the spinal muscles becoming hypertonic and sensitive with further muscle atrophy. Mechanical compression of spinal root and radiculopathy can also occur [4,5]. In young, sedentary populations, low back pain symptoms were associated with sustained sitting postures and increased flexion during relaxed sitting [6]. Moreover, spinal overuse, involving both daily occupational and sports activities, is responsible for the LBP. The harmful effects of both inactivity, sedentary control group behaviour, and strenuous levels of physical activity in sports active population were discovered [7,8].

The shape of the spine in a sitting position should be neutral, like the spine in stance, which is physiologically and biomechanically the best. The self-correction maneuver in standing and/or sitting position involves the entire top-down chain of motor control: cerebral cortex, somatosensory perception, muscle effectors, and motor output expressed by the central nervous system. Postural control is associated with the internal calibration and the internal image, where proprioception is involved in conscious and unconscious awareness of joint position sense, kinesthesia, and force sense of body parts [9]. However, the self-correction manoeuvre leading to a total improvement of standing natural erect posture is not instinctive and ideal, but it must be acquired with specific postural training. In general, asymptomatic, young participants were not able to focus and control their posture in an entire way but only on a few aspects at a time [10]. The same observation was discovered in patients with subacute and chronic non-specific LBP. They fail to focus and control their standing posture globally, showing better “attention” on posture to the sagittal plane but with substantial neglect of the lumbar spine [11]. Indeed, mild-moderate average pain intensity (3–6 points in NRS) in young participants with chronic LBP causes activation of nociceptors and may disrupt proprioceptive signals from muscle spindles, leading to reduced reliance on trunk proprioceptive signals for optimal posture control in balance tests [12].

When adopting a non-neutral sitting position, an asymmetric load on the spine appears with tensile and compressive forces incorrectly acting on the spine structures. Consequently, harmful muscle tension ensues, and pathological stress of the spine joints can occur [13]. When adopting a habitual passive sitting position, the pelvis is tilted back, which may exacerbate the pain in the lumbar spine [14]. Additionally, the lumbar section can lose its natural curvature, which may be flattened or even positioned kyphotically, which could lead to a lessening in muscle activity in the lumbosacral region and an increase in muscle activity in the neck area [15].

A sitting position reduces the angle in the lumbosacral region [16,17]. It can further cause overloads of the lower or upper region of the spine, which can result in the symptomatic pain. Prolonged sitting or standing in the workplace and improperly prepared workstations can lead to serious complications in the musculoskeletal system [13,18]. Sitting increases the passive stretching of the lumbar spine structures that can exacerbate the LBP [14]. Subjects with LBP are more sensitive to flexion and extension of the lumbar spine. A more neutral position of the spine is then recommended, including mild lumbar lordosis and a relaxed thoracic spine. Such a position is recommended to avoid the end-of-motion pain and provide more desirable activation of the trunk muscles [19,20].

According to the definition from the International Association for the Study of Pain, the pressure pain threshold (PPT) is the smallest mechanical stimulus that could cause pain sensations [21]. The PPT was also defined as the moment of pressure sensation when the actual pressure becomes both pressure and pain [22]. In young, healthy, asymptomatic individuals (*n* = 617), the neck was the most sensitive PPT test point, with the lower PPT more sensitive than the back and the leg pain point. Men were significantly less sensitive to pain, with higher PPT than women [23].

It is known that with higher levels of pain, peripheral and/or central sensitization of soft tissue begins [24]. Pressure pain threshold was the most predictive of patients with chronic non-specific low back pain [25], but the changes in the PPT observed in non-specific LBP might be mediated via gender differences [26]. The non-specific LBP group on average scored more than 3 (0–10 VAS scale), characterized by the widespread lower pressure and heat pain thresholds [27]. A moderate intensity of pain causes changes in the pain threshold and adaptations in the musculoskeletal system [24]. In order to investigate changes in the initial stage of the LBP dysfunction, the population of young people working in a sitting position but with low level of pain should also be investigated.

We therefore aimed to study postural alignments during sitting positions and overload symptoms in the PPT values over spinal muscles. The specific objectives of the study were: first, to compare the level of pressure pain threshold of soft tissue over the spine and the angles of spinal curvatures in habitual and corrected sitting positions in the low level of the LBP and control groups and, second, to identify physical factors associated with the low level of the LBP experienced by the young, white-collar workers.

## 2. Methods

### 2.1. Subjects

The study included office workers (*n* = 139), females (*n* = 51) and males (*n* = 88). They were divided into a control group (*n* = 82) and a group of individuals with non-specific pain in the lumbosacral region (*n* = 57) (Table 1).

The criteria for inclusion in the LBP group were as follows: (1) age of 25–35 years, (2) professional office work performed no less than six hours a day for a minimum of two years, and (3) non-specific chronic pain of 1–3 points on the Numeric Rating Scale (NRS) in the lumbosacral region on the day of the examination. The criteria for exclusion from the LBP group were neurological or orthopaedic conditions, specific back pain syndromes based on X-ray descriptions, diseases of the spine that qualify for surgery, pain at rest on the day of the examination indicating 4–10 points on the NRS scale, and pain, numbness, or tingling that radiates to the lower limb below the buttock fold.

The criteria for inclusion in the control group were (1) age of 25–35 years, (2) professional office work performed less than six hours a day for a minimum of two years, and (3) no back pain in the lumbar region in the last three months.

Before the initiation of the study, each subject was informed about the form of the study and about having the right to refuse to participate or withdraw at any time. The informed written consent was obtained from the study participants. The study procedures were approved by the Ethics Committee of the University School of Physical Education (25/2016).

### 2.2. Protocol

Measurements were performed by a single physical therapist possessing at least five years of experience in testing and body posture examinations. The subjects’ PPT was tested in a sitting position, using an FDIX RS232 algometer from Wagner (www.wagnerinstruments.com, accessed on 4 January 2022). The examination was performed at six measuring points, 2 cm from the spine on both sides at the apex of the cervical lordosis, the thoracic kyphosis, and the lumbar lordosis. The measuring tip of the algometer was placed perpendicular to the body and slowly pressed until the subject found the pressure to be unpleasant. The value (N/cm^2^) displayed on the device was recorded as the PPT. For a greater reliability, the measurements were made twice by the same person one minute apart, and the arithmetic average for each site was calculated [28].

The Numeric Rating Scale was used as a key instrument for pain intensity, and the Oswestry Disability Index (ODI) 2.1a (Polish version) was used to measure physical functionality in the studied LBP group. These are recommended as a core outcome measurement set to be included in every clinical trial in patients exhibiting the non-specific LBP [29].

The level of physical activity was determined using the short version of the International Physical Activity Questionnaire (IPAQ-SF). The questionnaire allows the study group to be classified into three groups according to their level of physical activity: high, medium, and low [30,31].

The curvatures of the spine were examined using the photogrammetric method based on two posture conditions: the habitual, normal or everyday sitting position (passive) and the corrected sitting position (self-corrected). The points were marked on the spinous processes of the vertebrae from C7 to S1, the thoracic–lumbar transition (T12/L1). The subjects sat with their back to the camera on a stool, the height of which was individually adjusted so that the feet rested freely on the ground, the bent knee angle was 90°, and the forearms rested on the tabletop. The distance from the camera was 2.6 m, and it was set so that the entire torso of the person being examined was visible on the monitor [32]. According to the American Society for Photogrammetry and Remote Sensing [33], photogrammetry is the science of obtaining reliable information about the shape of objects, which can be measured and interpreted. High inter-rater and test–retest reliability indices were found of photogrammetry in the measurement of the postural deviations [34]. A thorough understanding and evaluation of the ability to get a good performance in a self-correction posture are crucial in the prevention and rehabilitation of spinal disorders [10].

**Figure 1:**
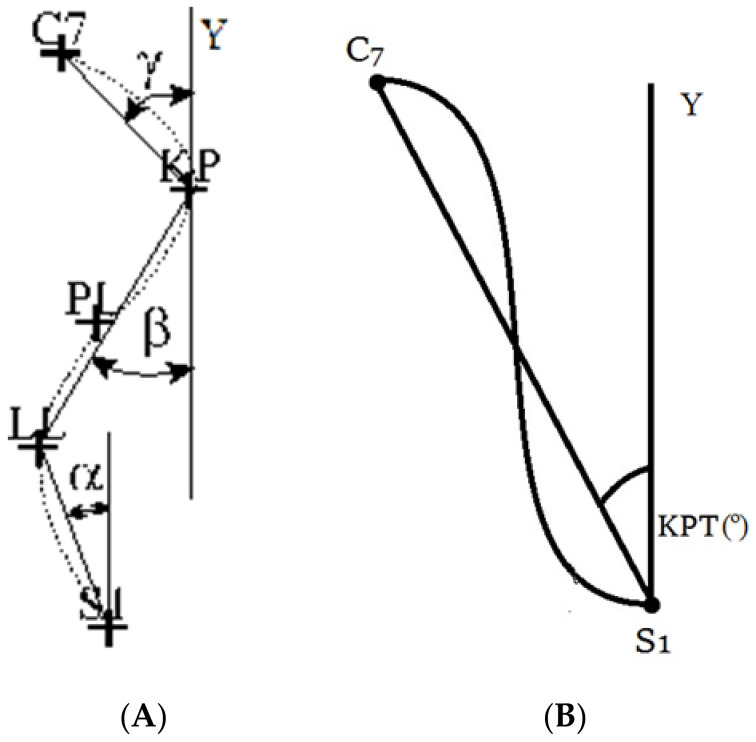
Angular parameters of the spine curvatures in the sagittal plane (**A**): the lumbosacral spine (α-ALFA), the thoracolumbar spine (β-BETA), the upper thoracic segment (γ-GAMMA), and torso angle (KPT) (**B**) [32].

The shape of the spine was recorded continuously for five seconds in the habitual position and five seconds in the self-corrected position with a frequency of 4 Hz. The instructions given to the subjects for the habitual position was “Please, adopt the sitting position which you usually maintain while working in the office”. For the corrected position, they were asked, “Please, correct your posture; sit down correctly”. The three-dimensional coordinates of the body surface were obtained, and the parameters determining the anterior-posterior curvature of the spine and the torso inclination in the sagittal plane were calculated. The following angular parameters of the spine curvatures were analysed: inclination of the lumbosacral spine (α-ALFA), inclination of the thoracolumbar spine (β-BETA), inclination of the upper thoracic segment (γ-GAMMA), and torso angle (Figure 1). Negative angle values indicated a forward tilt of the torso in relation to the vertical.

### 2.3. Statistics

The data were analysed using the statistical package Statistica 10.0 PL (StatSoft, Tulsa, OK, USA). Assuming a clinically significant effect size of the mean PPT 10 N/cm^2^ lower and a change of 2° in the angles of the spine, a sample size of 30 participants in each group was calculated to be sufficient to provide a study design with acceptable power (0.8) at *p* < 0.05. Since no statistically significant differences in the PPT were found between the measurements taken on the right and left sides of the body from both study groups, the results from both sides were then pooled and analysed together.

To evaluate the main effects and interactions of gender (women and men), group (LBP and control), or region (cervical, thoracic, and lumbar) on the PPT, multivariate ANOVA (2 × gender, 2 group × 3 regions) was conducted, followed by the Tukey test. Means and confidence intervals (CI) (±0.95) are presented in the figures. The anterior-posterior spinal curvature angles (ALPHA, BETA, and GAMMA) and the torso inclination angle were analysed by multivariate ANOVA (2 × gender, 2 × position, and 2 × group). The main factor effects and interactions were calculated, and post hoc testing with the Bonferroni test was then performed.

The logistic regression analysis was performed separately for the groups of men and women. In the model, the dichotomised LBP (categorised into no pain (NRS = 0) and the low-level LBP (NRS = 1–3)) were entered as a dependent variable, and the continuous measurements were the independent variables. The odds ratio (OR) with ±95% confidence interval (CI) was estimated, indicating associated variables with the probability of the low level. The levels of significance were set at * *p* < 0.05, ** *p* < 0.01, and *** *p* < 0.001.

## 3. Results

### 3.1. Demographic and Descriptive Statistics

The comparison of demographic data between the LBP and the control groups revealed a significant difference in the body mass of women (*p* < 0.05). The remaining demographic data did not differ significantly between the LBP and the control groups (Table 1).

### 3.2. Pressure Pain Threshold

There was main effect of gender (F_(135)_ = 53.37, *p* < 0.001). In the post hoc tests, female white-collar workers had lower PPTs than the male subjects (*p* < 0.001), with the exception of the PPT in the cervical part (*p* = 0.09). There was a main effect in spinal regions (F_(270)_ = 333.11, *p* < 0.001) and statistical interaction between gender and spinal regions (F_(270)_ = 54.54, *p* < 0.001). In the post hoc tests, there were significant differences between the PPTs in the cervical, thoracic, and lumbar areas among the men (*p* < 0.001), but in the group of women, there were no differences between the PPTs in the thoracic and lumbar parts (*p* = 0.06).

**Figure 2:**
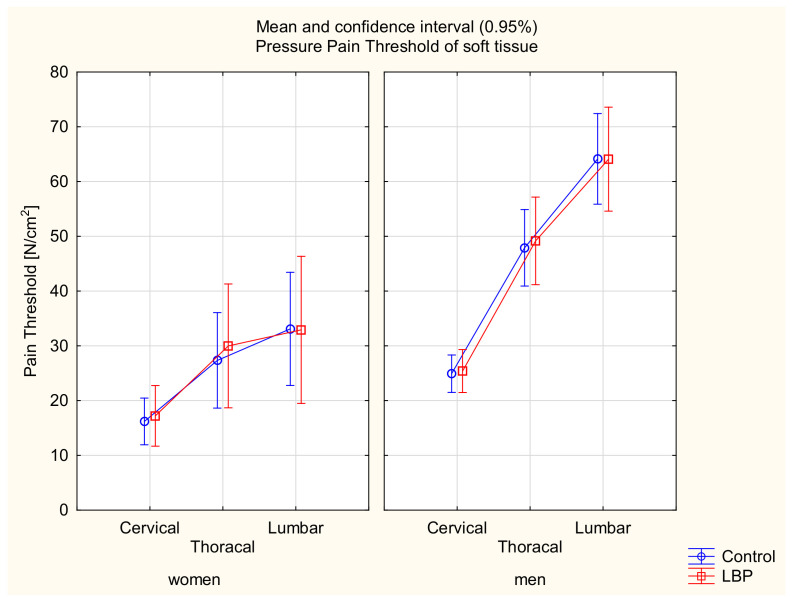
Mean and 95% CI of the pressure pain threshold in women and men groups and in the control and LBP groups.

There were no differences between the asymptomatic and symptomatic subjects within the women and men groups in the three tested regions of the spine (F(_270)_ = 0.06, *p* > 0.05) (Table 1) (Figure 2).

### 3.3. Angular Parameters of the Spine Curvatures

#### 3.3.1. ALPHA Angle of the Lumbosacral Spine

There were main effects in the gender (F_(__135)_ = 41.822, *p* = 0.001) and the sitting position (F_(__135)_ = 15.413, *p* = 0.001) and a statistically significant interaction between the two factors (F_(__135)_ = 21.269, *p* = 0.001).

In the post hoc test, a statistically significant difference was found in the ALPHA angle between the habitual and corrected position only among women from the control group (*p* < 0.001), and an insignificant difference was found in the LBP group (*p* = 0.06). There were no differences in the ALPHA angle between the habitual and corrected positions in the LBP group among men (*p* > 0.05). There were also no differences between the groups in either position (*p* > 0.05).

**Figure 3:**
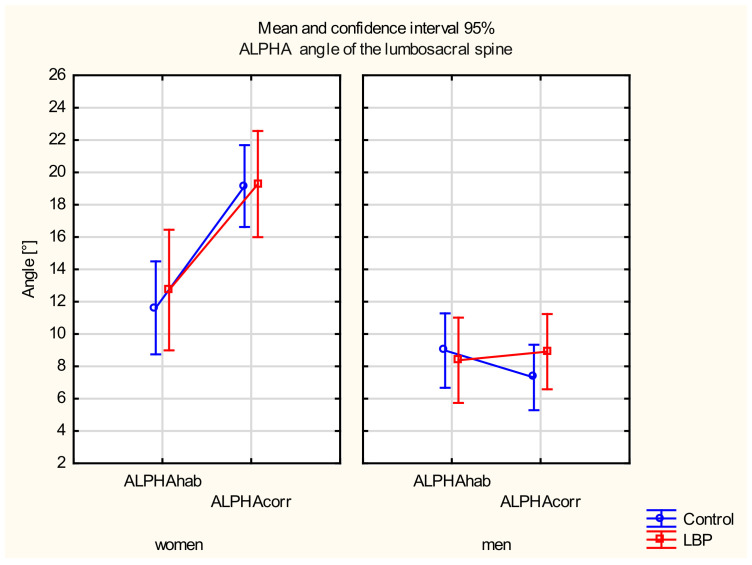
Mean and 95% CI of the ALPHA angle of the lumbosacral spine in women and men in habitual (hab) and corrected (corr) positions, in the control and LBP groups.

A statistically significant difference was found in the ALPHA angle in the corrective position between women and men from the control group (*p* < 0.001) and the LBP group (*p* < 0.001). The women displayed a significantly higher ALPHA angle in the corrected position (Figure 3).

#### 3.3.2. BETA Angle of the Thoracolumbar Spine

There were main effects of the gender (F_(__135)_ = 10.281, *p* < 0.01) and the sitting position (F_(__135)_ = 93.790, *p* < 0.001) as well as a statistically significant interaction between the two factors (F_(__135)_ = 5.1274, *p* < 0.05). There was also a statistically significant interaction between the gender and the pain factor (F_(__135)_ = 11.001, *p* < 0.001) and between the three factors: gender, pain, and the sitting position (F_(__135)_ = 8.8553, *p* < 0.01).

**Figure 4:**
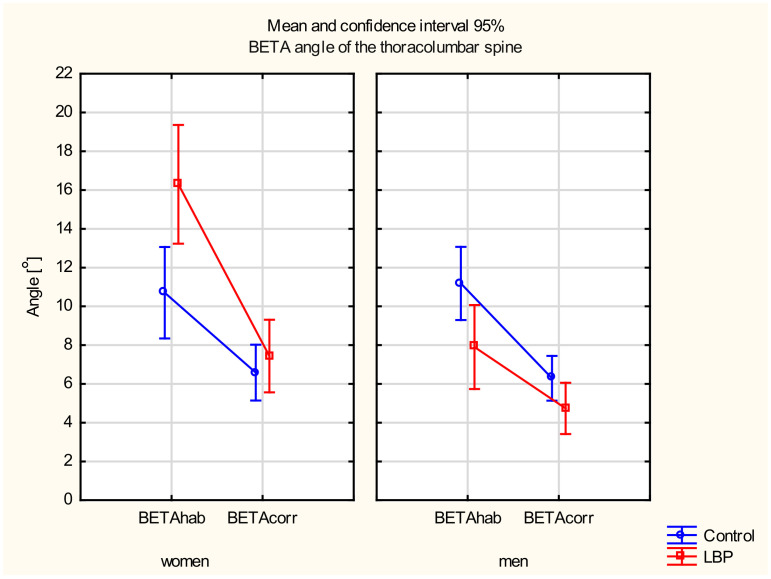
Mean and 95% CI of the BETA angle of the thoracolumbar spine in women and men, in habitual (hab) and corrected (corr) positions, in the control and LBP groups.

In the post hoc test, a significant decrease in the BETA angle between the habitual and the corrected sitting position was found in the LBP group in both women (*p* < 0.001) and men (*p* < 0.05). A statistically significant difference in the BETA angle between the two positions also occurred in the control group of women (*p* < 0.01) and men (*p* < 0.05). When comparing the study groups, a statistically significant difference occurred only in the BETA angle in the habitual sitting position in women (*p* < 0.05). The women in the LBP group were characterised by a significantly higher BETA angle. There were no differences in the BETA angle between the control and LBP groups in either position in men or women in the corrected sitting position (*p* > 0.05). A statistically significant difference was found in the BETA angle in the habitual sitting position between women and men from the LBP group (*p* < 0.001) (Figure 4).

#### 3.3.3. GAMMA Angle of the Upper Thoracic Spine

**Figure 5:**
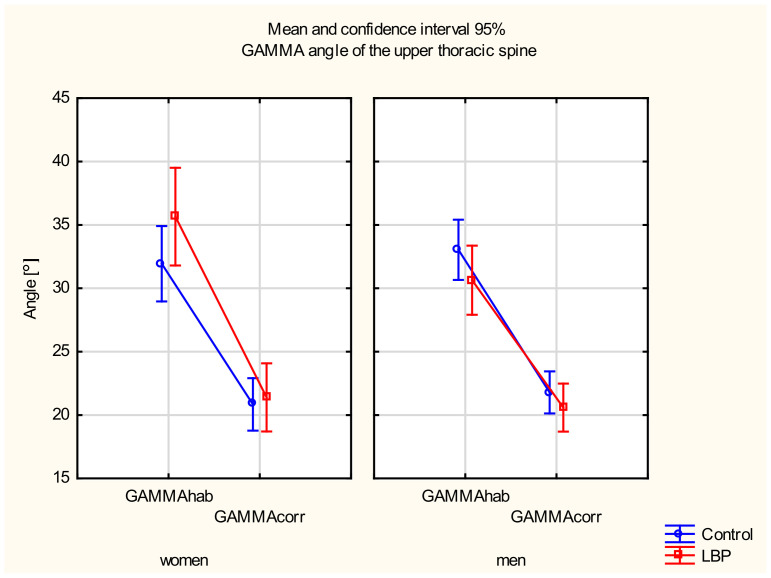
Mean and 95% CI of the GAMMA angle of the upper thoracic spine in women and men, in habitual (hab) and corrected (corr) positions, in the control and LBP groups.

A main effect of position was observed (F_(__135)_ = 359.51, *p* < 0.001). No main factor effect between the gender and the level of pain was observed (*p* > 0.05). In the post hoc test, a significant decrease in the GAMMA angle between the habitual and the corrected sitting positions in the LBP group was found in both women (*p* < 0.001) and men (*p* < 0.001). A statistically significant difference in the GAMMA angle between the two positions also occurred in the control group in both women (*p* < 0.001) and men (*p* < 0.001). There were no differences in the GAMMA angle between the control and the LBP groups in either position (*p* > 0.05) (Figure 5).

#### 3.3.4. Torso Angle

There were main effects of the gender (F_(135)_ = 19.437, *p* < 0.001) and the sitting position (F_(135)_ = 119.23, *p* < 0.001) and a significant interaction between these two factors (F_(135)_ = 5.3137, *p* < 0.05). There was also a statistically significant interaction between the gender and the pain factors (F_(135)_ = 7.4166, *p* < 0.01).

**Figure 6:**
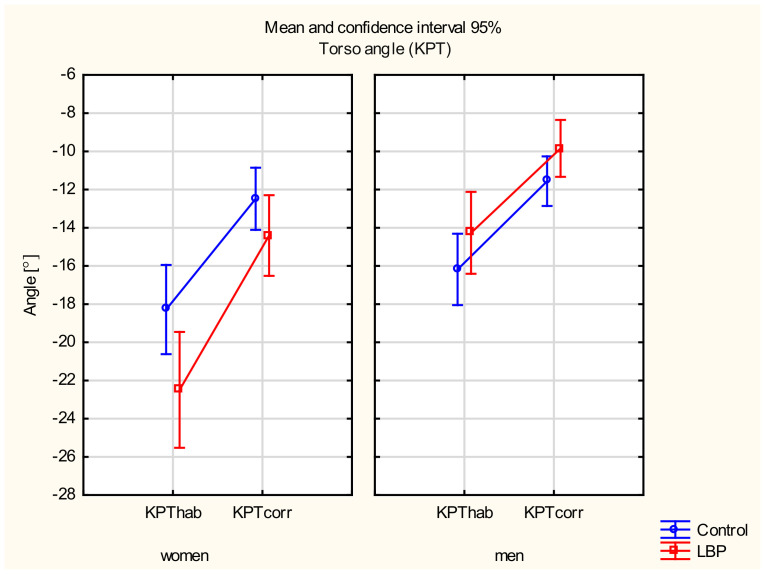
Mean and 95% CI of the torso angle in women and men, in habitual (hab) and corrected (corr) positions, in the control and LBP groups.

In the post hoc test, a statistically significant difference was found in the torso angle between the habitual and the corrected sitting positions in the LBP group in both women (*p* < 0.001) and men (*p* < 0.001). A statistically significant difference between these variables was also found in the control group in both women (*p* < 0.001) and men (*p* < 0.001). There were also no differences in the torso angle between the control and the LBP groups in either position (*p* > 0.05). A statistically significant difference occurred in the habitual sitting position in the LBP groups between women and men (*p* < 0.001) (Figure 6).

### 3.4. The Logistic Regression Model

In the base model, the dichotomised LBP (categorised into no pain (NRS = 0) and low pain (NRS = 1–3)) was entered as the dependent variable and the continuous measurements taken were the independent variables. Pressure pain threshold over thoracic and lumbar spine and the angle of the thoracolumbar in the habitual sitting position (BETA_hab_) were associated with low back pain only in females. The BMI and angles of the spine in the corrected sitting position—ALFAcorr, GAMMA_corr_, and KPT_corr_—were associated in males (*p* < 0.15) (Table 2).

The following variables were included in the final logistic regression model but only in the group of women: the PPT over the thoracic spine (OR/CI = 1.10/1.02–1.19), the lumbar spine (OR/CI = 0.90/0.83–0.98), and the angle of the thoracolumbar in the habitual sitting position (BETA_hab_) (OR/CI = 1.19/1.05–1.34) were associated with low back pain in females, (*p* < 0.05) (Table 2).

The following variables were included in the final logistic regression model in the group of men: the angle of the torso in the corrected sitting position (KPT_cor_) (OR/CI = 1.14/1.01–1.29) (*p* < 0.05), the angle of the lumbosacral spine in the corrected sitting position (ALPHA_cor_) (OR/CI = 1.06/0.98–1.15), and BMI (OR/CI = 1.56/0.84–2.90) (*p* > 0.05). The model for men showed a significant probability for the angle of torso in the corrected sitting position (KPT_cor_) only (Table 2).

## 4. Discussion

In brief, the most interesting findings were that the PPT of soft tissue in the spinal region of the low-level LBP group was similar to the control group but significantly lower in women than men, with differences between regions; there were no differences in the ALPHA angle between the habitual and the corrected sitting positions in the LBP group but only among men; a difference between the LBP and the control groups occurred in the BETA angle in the habitual sitting position and only among women; physical risk factors associated with low-intensity back pain in young, white-collar workers were identified but differed between women and men.

### 4.1. The Pressure Pain Threshold

The significant differences were observed in the PPT in all regions of the spine between women and men in both the LBP and the control groups. This observation is consistent with the literature, as generally women have a lower level of the PPT than men [35], and the effect of gender decreases with age [25]. The values reported in our study were within the reference values of the healthy population for the lumbar region in both women (mean(SD) = 38.2(17.9) N/cm^2^) and men (mean(SD) = 54.1(24.1) N/cm^2^), while for the cervical region in both sex groups, they were within the range of the proposed reference PPT with reduced sensitivity (mean = 15.5–21.4 N/cm^2^) [23].

No significant differences were found in the PPTs between the LBP and the control groups in both the women and men. In other studies, lower PPTs values were found in people with LBP compared to healthy people [36], but in patients with mechanical low back pain, the PPTs did not differ from the controls, with two distinguished non-specific low back pain subgroups (26). The conflicting presented results may be due to the fact that only subjects with low pain intensity (1–3 points on the NRS scale) were qualified for the study. It can be interpreted that there is a certain level of intensity of pain at rest from which the PPT value changes, and sensitization of the soft tissue could develop with a higher level of pain at rest as a secondary symptom [24]. Spinal structures could be sensitized to movement and/or load but not local pressure. The point where the PPT testing was applied was not specific to their pain location, and thus, probably sensitization of passive lumbar structures could occur.

### 4.2. Angular Parameters of Spine Curvature

There were no differences in the ALPHA angles between the habitual and the corrected siting positions in the LBP group in men. Significantly higher ALPHA angles in the corrected sitting position were found in women than men. This observation indicates a greater ability to correct or increase this angle by women compared to men. In contrast, another study found no correction in the lumbar spine region of asymptomatic women [32].

There were differences in the lumbosacral spine and pelvis position between women and men [37,38]. In the sitting position, women were characterised by a greater angle of lumbar lordosis and sacrum than men; these differences may result from the differences between pelvic skeletal structure between women and men [38]. However, in our research, no differences were found between the LBP group and the control group. This observation indicates that the lumbosacral angle of the spine in a sitting position was not forced in the LBP subjects, but men from the LBP group did not effectively correct the lumbosacral angle.

It was found that people with LBP did not differ from asymptomatic people with regard to the angle of lumbar lordosis or the angle of the anterior tilt of the pelvis in a standing position although they did show differentiation in terms of the range of motion of the lumbar spine in all directions, the velocity of movement of this section, and worsened proprioception during movement reposition [39]. These observations emphasise the disturbances in the motor function of the lumbar spine [40] rather than changes in the shape of the anterior-posterior curves in people with the LBP. The presented study confirms that the lumbosacral angle did not differentiate between the study groups.

In another review, patients with disc pathology were shown to have a lower lumbosacral angle than those without pain [41]. Therefore, changes in the shape of the spine in the lumbar region may be secondary and adaptive to pathological changes in the advancement of disorders in the structure of the spine, which requires further research. In our study, the LBP group included subjects with low-intensity, nonspecific pain syndrome and no disc pathologies; the tests concerned only the shape of the anteroposterior curves in a sitting position, hence the discrepancy of the results.

Comparing the control and LBP groups, a statistically significant difference occurred only in the BETA angle in the habitual sitting position in women. The adoption of habitual sitting postures gave rise to a kyphotic posture in asymptomatic women [32]. This finding corresponds to O’Sullivan’s slump sitting, in which the thoracolumbar spine was relaxed [42]. In studies of the asymptomatic subjects, it was found that the short-term (20 min) adoption of a slouched, corrected, or supported sitting position had no effect on the intensity of pain at rest, range of motion, muscle activity in the lumbar spine, or proprioception [43]. However, long-term sitting in a lordotic or kyphotic sitting position may be an important factor causing and maintaining pain in the lumbar region of the spine [44]. Low back muscle activity was very limited while seated, presumably due to the flexion–relaxation phenomenon, which occurs in a kyphotic posture. The altered flexion–relaxation phenomenon was found in about half of the non-specific LBP population, and this prevalence was much greater than in the asymptomatic adult population [45].

According to the opinion polls of the Greek physiotherapists (*n* = 544), in the optimal sitting position, the shape of the lumbar lordosis should be similar to the shape of this curvature in a standing position, with thoracic kyphosis and head positioning in retraction rather than protraction [44]. Adopting such a posture also depends on corrective skills, which were different in women and men. The ability to correct the angle of the thoracolumbar spine, the angle of the upper thoracic segment, and the angle of the torso were identified and in both LBP groups; however, only men did not significantly correct the lumbosacral angle of the spine.

### 4.3. The Logistic Regression Model

The following variables were associated with the low back pain but only in females: the PPT over the thoracic spine and the lumbar spine and the angle of the thoracolumbar in the habitual position (BETA_hab_).

These observations need to be emphasised in an active sitting position; correction concerns the positioning of the pelvis in a forward tilt and thus adopting a lordosis shape both in the lumbar and cervical sections while maintaining a slight thoracic kyphosis. As a result, a balanced posture should dominate. Maintaining an active sitting position aligns the spine near the neutral zone and, as a result, helps to maintain the pain threshold in the lumbar and the thoracic area at an optimal level [46]. The meaning of the PPTs was confirmed by Gupta et al.: the tender points count and pressure pain threshold might be predictive factors of chronic pain [47].

Among men, angles of the torso and the lumbosacral spine in corrected-active siting position and the BMI were associated with low-level LBP. This implies that proposed strategies at work should be investigated for the re-education of the sitting posture. 

There was consensus for the absence of an association between exposure to prolonged or occupational sitting and the LBP, but with respect to the other physical exposures examined, including sagittal spine curvatures, prolonged or occupational standing, awkward postures, bending and twisting movements of the spine, components of heavy physical work, and whole-body vibration, the evidence was conflicting [48]. When considering meta-analyses alone, consistent, significant, and positive associations were demonstrated between maintaining flexed and non-neutral postures and the LBP [49]. Our own study underlined the flexed spinal posture and pressure pain threshold as associate risk factors in the low-intensity back pain but only in females. Furthermore, self-corrective skills in a sitting position may be one of the easiest to implement exercises. Re-educated, self-corrected posture with specific postural training would be expected to improve proprioception in postural control. There is preliminary evidence that a single session of proprioceptive neuromuscular facilitation exercises has a beneficial effect to alleviate pain in chronic LBP patients [50].

Limitations: The sample size in the LBP women group was relatively small; low-intensity pain as an inclusion criterion could not be discriminative in the LBP subjects; the IPAQ questionnaire was used, which is quite a subjective tool for assessing the level of physical activity; sagittal spine curvatures were measured indirectly; the pelvic angle was not measured although this variable was found to correlate most to low back muscle activity in a sitting position [15] and could affect spinal curvatures. There were no distinguished two non-specific low back pain subgroups: mechanical and non-mechanical [26], and this aspect could be studied in the next perspective.

## 5. Conclusions

White-collar workers with low-intensity, non-specific back pain characterized similar PPTs of the soft tissue to the asymptomatic subjects. In the multifactorial nature of the LBP, habitual, flexed sitting posture and the pressure pain threshold may be considered as predictive factors of low back pain in female office workers. Men from the LBP group did not effectively correct the lumbosacral angle. Therefore, re-educated, self-corrected posture with specific postural training would be expected to improve proprioception in postural control capacity and result in decreasing pain. The health behaviour strategies while conducting office work should be investigated to re-educate the correct sitting postures to alleviate the back pain as much as possible. The presented cross-sectional study is not conclusive, so the randomized control trials should be studied with individually tailored preventive programs focused on maintaining optimal posture and self-correction of sitting position in the white-collar worker population.

## Figures and Tables

**Table 1:** Demographic.

	LBP (*n* = 57)		CONTROL (*n* = 82)		*p*-Value LBP/CON Females	*p*-Value LBP/CON Males
	F (*n* = 19) Mean (SD)	M (*n* = 38) Mean (SD)	F (*n* = 32) Mean (SD)	M (*n* = 50) Mean (SD)		
Age (years)	29.00 (3.68)	29.39 (3.16)	29.59 (3.42)	29.72 (3.00)	0.56	0.63
Mass (kg)	64.42 (9.27)	86.95 (13.67)	59.31(7.75)	83.20 (11.95)	0.03 *	0.17
Height (m)	1.67 (0.07)	1.80 (0.06)	1.66 (0.05)	1.80 (0.06)	0.64	0.87
BMI	23.11 (3.75)	26.67 (4.0)	21.39 (2.72)	25.56 (3.27)	0.06	0.15
T_Y_ (years)	5.47 (3.39)	6.21 (2.87)	6.03 (3.30)	6.02 (3.08)	0.56	0.77
T_D_ (hours)	10.11(1.70)	9.84 (1.94)	9.44 (1.92)	9.70 (1.96)	0.21	0.73
Insuff PA (*n*)	3	6	5	8	Chi^2^ = 0.06, *p* = 0.80	
Suff PA (*n*)	11	19	18	29	Chi^2^ = 0.02, *p* = 0.88	
High PA (*n*)	5	13	9	13	Chi^2^ = 0.75, *p* = 0.39	
NRS	1.95 (0.78)	2.23 (0.79)	-	-	0.19	
ODI	9.10 (5.95)	7.07 (4.83)	-	-	0.17	
PPT cervical	17.21 (5.25)	25.4 (11.22)	16.20 (6.10)	24.92 (9.07)	1.0	1.0
PPT thoracic	29.99 (7.66)	49.16 (24.81)	27.35 (9.92)	47.90 (18.56)	0.99	1.0
PPT lumbar	32.93 (7.14)	64.09 (28.32)	33.10 (11.79)	64.15 (23.19)	1.0	1.0

Abbreviations and symbols: control group (CON), low back pain group (LBP), females (F), males (M), insufficient/sufficient physical activity (Insuff/Suff PA,) numbers (*n*), standard deviation (SD), chi-square (Chi^2^), working time in years (T_Y_), daytime in sitting position (T_D_), numerical rating scale (NRS), Oswestry Index (ODI), pressure pain threshold (PPT), level of significance (* *p <* 0.05).

**Table 2:** The logistic regression model estimation in females and males considering low back pain (NRS < 3) as dependent variable.

Model Steps	Variables	Females (*n* = 51)		Males (*n* = 88)	
		OR (95% CI)	*p*	OR (95% CI)	*p*
Base model ^a^					
	BMI	1.89 (0.3–13.64)	0.51	1.8 (0.9–3.64)	0.08 *
	PA	1.56 (0.32–7.68)	0.56	1.46 (0.67–3.16)	0.32
	T_Y_	0.90 (0.71–1.16)	0.42	1.05 (0.88–1.24)	0.56
	T_D_	0.96 (0.59–1.57)	0.88	1.12 (0.86–1.46)	0.36
Pressure pain threshold					
	Cervical	1.02 (0.88–1.16)	0.80	0.99 (0.97–1.02)	0.93
	Thoracic	1.1 (0.99–1.22)	0.06 *	1.00 (0.98–1.01)	0.90
	Lumbar	0.90 (0.81–0.98)	0.03 *	1.10 (0.97–1.11)	0.91
Habitual position					
	ALFA_hab_	0.99 (0.83–1.18)	0.96	0.97 (0.88–1.07)	0.65
	BETA_hab_	1.30 (0.91–1.85)	0.13 *	0.97 (0.83–1.13)	0.72
	GAMMA_hab_	1.02 (0.68–1.54)	0.89	0.96 (0.80–1.16)	0.72
	KPT_hab_	1.14 (5.3–2.40)	7.25		
Corrected position					
	ALFA_corr_	0.96 (0.82–1.13)	0.66	1.06 (0.97–1.17)	0.15 *
	BETA_corr_	0.86 (0.56–1.33)	0.49	0.99 (0.83–1.17)	0.93
	GAMMA_corr_	1.03 (0.69–1.54)	0.85	1.16 (0.95–1.42)	0.13 *
	KPT_corr_	9.12 (4.41–1.88)	7.97	1.36 (0.97–1.88)	0.06 *
**Final model** ^**b**^					
	PPT_Th_	1.10 (1.02–1.19)	0.009 **	-	-
	PPT_L_	0.90 (0.83–0.98)	0.01 **	-	-
	BETA_hab_	1.19 (1.05–1.34)	0.002 **	-	-
	BMI	-	-	1.56 (0.84–2.90)	0.14
	KPT_corr_	-	-	1.14 (1.01–1.29)	0.02 *
	ALFA_corr_	-	-	1.06 (0.98–1.15)	0.10

^a^ Base model: variable associated (* *p* < 0.15); ^b^ Final model: statistically significant results (* *p* < 0.05, ** *p* < 0.01); Abbreviations and symbols: numerical rating scale (NRS), odds ratio (OR), confidence interval (CI), body mass index (BMI), physical activity (PA), working time in years (T_Y_), day time in sitting position (T_D_), pressure pain threshold (PPT) over cervical (C), thoracic (TH) and lumbar (L) spine, inclination of the lumbosacral spine (ALFA), inclination of the thoracolumbar spine (BETA), inclination of the upper thoracic segment (GAMMA), torso angle (KPT), habitual position (_hab_), corrected position (_corr_).
